# The Effect of Assistive Anchor-Like Grousers on Wheeled Rover Performance over Unconsolidated Sandy Dune Inclines

**DOI:** 10.3390/s16091507

**Published:** 2016-09-15

**Authors:** Ahmad Najmuddin Ibrahim, Shinichi Aoshima, Naoji Shiroma, Yasuhiro Fukuoka

**Affiliations:** 1 Graduate School of Science and Engineering, College of Engineering, Ibaraki University, Hitachi 316-8511, Japan; 13nd207t@vc.ibaraki.ac.jp; 2 Department of Intelligent Systems Engineering, College of Engineering, Ibaraki University, Hitachi 316-8511, Japan; shinichi.aoshima.blue@vc.ibaraki.ac.jp (S.A.); naoji.shiroma.iu@vc.ibaraki.ac.jp (N.S.)

**Keywords:** wheeled rover, traversing sandy dune incline, assistive grousers

## Abstract

Typical rovers with wheels equipped with conventional grousers are prone to getting stuck in unconsolidated sandy dune inclines as the wheels tend to sink into the sand. This phenomenon is caused by the motion of the grouser through the sand during the latter half of the rotation, in which the grouser pushes the sand from underneath the wheel upwards and towards the backside of the wheel. This creates a space that the wheel can sink into. To minimize sand movement and subsequent sinkage, we propose the concept of using an “assistive grouser”, which is attached to the side of a conventional rover wheel. The assistive grouser is designed to be able to autonomously maintain a uniform angle relative to the rover body independent of the rotation of the wheels. Rotating the wheel causes the assistive grousers to automatically penetrate into the sand slope surface at a constant angle of attack, thereby acting as an anchor and providing traction for the wheel. Maintaining a uniform grouser angle as opposed to a rotating motion also assists in extracting the grouser out of the sand without moving the sand towards the back of the wheel. Moreover, the angle of the assistive grousers is held constantly by a single dedicated motor, meaning that the angle of the assistive grousers can be optimized to provide the least amount of sinkage for each slope angle. The experimental results showed that for slope angles of 0–30 degrees, the rover equipped with the proposed assistive grousers experienced significantly less sinkage and consumed less current compared to the rover equipped with conventional grousers.

## 1. Introduction

Wheeled rovers have a long history, and their mobility has been an issue garnering extensive research and interest over the years. Recently, much research has focused on improving mobility performance on one of the most challenging terrains to traverse for the outdoor wheeled rover; the unconsolidated sandy incline. The importance of controlling the amount of sinkage when traversing on sandy inclines was proven when the planetary rover “Spirit” Mars rover was embedded into the sand and unable to move forward when moving on a sand slope in 2009 [1]. All extrication plans to free it has failed. Its twin rover “Opportunity” was also immobilized and the wheels sunk into the sandy terrain. Opportunity however, managed to recover from getting stuck in the Purgatory Dune in 2005 after 5 weeks of continuous effort [2]. Efforts to improve mobility on sandy slopes have been made [3,4,5,6,7,8,9], but traversing steep slopes with low levels of sinkage has proven to be difficult. In particular, no successful attempt has yet been recorded for traversing on sand surfaces inclined at the angle of repose (the maximum angle of the slope face created by a flowing granular material when it is piled onto itself before the material slides under its own weight; e.g., approximately 30 (29.71) degrees for Toyoura fine sand [10]. This research aims to successfully traverse a steep slope of loose sand with low levels of sinkage using a wheeled rover, including even at the angle of repose. We believe that a complex mechanical system with a large degree of freedom, requiring a special control method, may not be widely used and therefore, we propose the use of an optional assistive grouser mechanism that is attached to a conventional rover wheel to aid its mobility on soft sand terrain. When a conventional wheel is better suited to the task, the assistive grousers’ angles can be configured such that it does not interfere with the wheel contacting the ground, thereby increasing the rover’s versatility to beyond that of sandy terrains.

The effects of loose sand on rigid wheel mobility have been widely investigated, and several parameters have been found to affect the performance of rover wheels. Sutoh et al. reported that increasing rover weight will decrease its traveling performance, and that installing grousers on the wheel is more effective in improving mobility than increasing the wheel diameter and width [5]. Similar findings were reported by Ding et al., which also added that the wheel-soil interaction mechanics is little influenced by the number of repetitive wheel passing on the same path and the wheel velocity for a low-speed rover [11]. Nakashima et al. investigated the effects of wheel parameters (diameter, width) and grouser parameters (number attached, height, thickness) on wheel performance through simulation and experiments using a sloped mobility test bed. They reported that increasing the height of the grouser and increasing the number of grousers attached to the wheel will increase both the gross traction and running resistance during slope locomotion [12]. Yamamoto et al. recorded grouser-soil interaction forces when a grouser is rotated beneath a soil surface, and concluded that a longer grouser sinkage length will generate a larger peak force. They also found the relationship between the force and maximum grouser sinkage length can be approximated using a quadratic linear function [13]. Iizuka et al. also demonstrated that a larger wheel diameter and longer grouser length generate less slippage on slopes of loose soil [14]. Hermawan et al. [15] and Yang et al. [16] proposed mechanisms to adjust grouser sinkage length and grouser contact angle with the soil, and reported improvement to soil-wheel interaction forces compared to conventional fixed grousers.

### 1.1. Conventional Fixed Grousers

**Figure 1:**
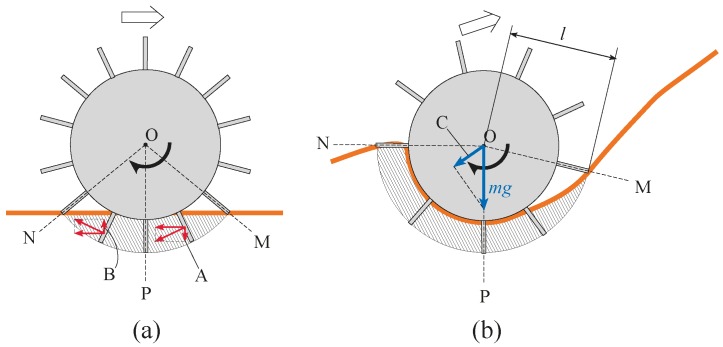
Conventional rover wheel: (**a**) On a level incline; (**b**) On a steep incline.

Attaching grousers to the wheel helps to gain traction to move forward on sandy terrain; however in flowing unconsolidated sand inclines, conventional grousers are not always considered effective. To take a specific example, the grouser and wheel setup shown in Figure 1a is able to gain traction to move forward from the grouser movement between the point of grouser entry into the sand (OM) to the vertical line (OP), which directs force A downwards pushing the sand and stiffening the local sand region. However, the grouser movement from OP to the point where the grouser exits the sand (ON) directs force B upwards, pushing the sand from below the wheel upwards and lifting it towards the surface. The amount of sand lifted from under the surface increases with the slippage of the wheel. Increasing the grouser length will increase the traction force obtained from the OM-OP grouser movement, but the volume of sand moved from below the surface by grouser movement OP-ON also increases. On a level and a moderate surface incline, the force of gravity component parallel to the surface (force C in Figure 1b) is small, and so the traction force produced by the OM-OP grouser movement dominates the OP-ON grouser movement induced sand movement, resulting in less slipping and sinkage with longer grouser length. Generally, however, for steep inclines such as that shown in Figure 1b, the sand in front of the wheel crumbles and flows downwards, and a large volume of sand will accumulate behind the wheel. Consequently, the volume of sand moved by the OP-ON grouser movement (marked by the shaded area in Figure 1b) will be significantly larger than that on a level or moderate surface incline. The volume of sand moved by the grouser proportionally increases with grouser length while it is difficult for even longer grousers to gain enough traction from the OM-OP grouser movement over steep inclines because of the large force C (Figure 1b). A higher torque output is also needed with a longer grouser length after the grouser has entered the sand and passed line OM. On the other hand, a shorter grouser length causes less sand movement, but the amount of traction force generated is generally not enough to drive the wheel forward, resulting in slippage as the wheel turns and the accumulation of large volume of sand at the backside of the wheel. For these reasons, traversing steep soft sand inclines with low levels of sinkage and a low probability of getting stuck are difficult using wheels equipped with conventional grousers, regardless of the grouser length.

### 1.2. Proposed Assistive Grousers

**Figure 2:**
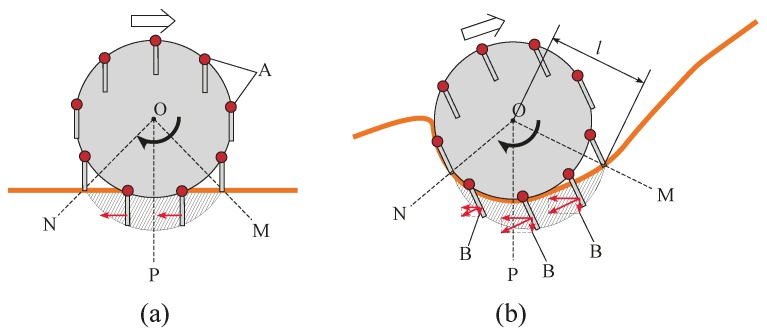
Grouser at a constant angle: (**a**) On a level incline; (**b**) On a steep incline.

Grousers are generally fixed to the wheel in a rigid manner, with the result that the contact angle of the grouser with the sand surface constantly changes as the wheel rotates. As seen in Figure 1, this causes the grouser to lift up sand from below the wheel during the OP-ON grouser motion. In this paper, we propose the concept of anchor-like assistive grousers (Figure 2) which are able to maintain a uniform angle independent of wheel rotation, in order to improve the performance of wheels on loose sand inclines.

**Figure 3:**
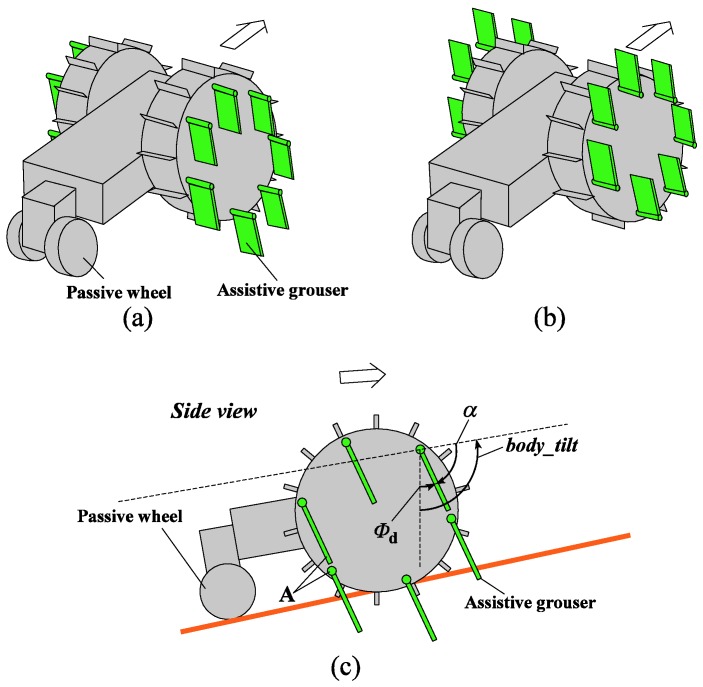
Diagram of a wheeled rover with assistive grouser mechanism: (**a**) Assistive grouser angle pointing downwards to be in contact with the terrain; (**b**) Assistive grouser angle rotated upwards away from ground surface; (**c**) Relationship between *body_tilt*, *α* and *Φ_d_*.

These grousers are attached to shafts, labeled A in Figure 2a and rotate in sync as the shafts are mechanically connected to one another. As seen in Figure 2a, if the assistive grouser angle is set so as to maintain an angle parallel to the direction of the gravitational force as the wheel turns, the OP-ON grouser movement does not lift any sand towards the surface, reducing the amount of sand accumulated behind the wheel. As the grouser angle is maintained vertical relative to the sand surface, horizontal traction force is constantly gained as the rover moves forward. This mechanism is especially effective on slopes of soft sand, as seen in Figure 2b, if the grouser angle is maintained at an angle slightly in front of the gravitational force direction. It can be seen that more traction force is gained through movement OM-OP moving the sand in the shaded area. While the grousers are in contact with the sand, downward force B also helps to climb the slope. Conversely, the shaded sand volume during grouser movement OP-ON is smaller, and furthermore, the constant grouser angle means that the grouser will exit the sand surface smoothly rather than lifting a large amount of sand with it. In light of this, we believe that a rover so equipped will be able to traverse slopes without accumulating large amounts of sand behind the wheel. By virtue of these special characteristics, we assume that elongating the length of the grousers will generate a larger traction force while not increasing the amount of sand shifted from beneath the wheel toward the back. When conventional grousers enter the sand at OM (Figure 1b), a longer grouser length will generate a longer moment arm, *l*, necessitating a larger torque to turn the wheel. Our proposed assistive grousers, however, enter the sand surface through a piercing-like motion (Figure 2b), creating a shorter moment arm, *l*, and so reducing the amount of torque needed to turn the wheel. We expect that even large assistive grouser lengths can pierce the sand in the same manner and act as an effective anchor for the rotating wheel, reducing slippage and helping the rover to move up steep inclines. The grouser-soil interaction between the sand and the assistive grousers however, is more complex than from only piercing motion alone, and this is discussed in Section 5.2. We created a simple wheeled rover prototype (Figure 3) and mounted it with our proposed assistive grouser mechanism, as shown in Figure 2. The rover was designed as an experimental tool to examine the effectiveness of our proposed assistive grouser; therefore, only the two front wheels were driven wheels, and the rear wheel was a passive wheel that rotated with minimal resistance. We chose to carry out experiments using a real rover instead of a numerical simulation, as the sand-grouser interaction of the conventional grouser and our proposed assistive grouser is difficult to model. In the future, we hope to also investigate the use of assistive grousers on four and six wheel driven rovers. Assistive grousers are mounted to the side of a wheel equipped with a conventional fixed grouser as an optional assistive mechanism to avoid the problem of the assistive grousers rotating into the wheel surface itself when positioned inside the wheel (see Figure 3a). Using an assistive grouser that is longer than the length of the fixed grouser will help the rover to climb steep soft sand inclines, but when the long assistive grouser is not needed or would interfere with the movement of the wheel, such as when the rover traverses hard surfaces, the assistive grousers can be rotated away from the ground, as shown in Figure 3b. This allows the wheel to work as a normal wheel even when equipped with the assistive grouser, increasing the versatility of the grouser.

To realize this concept, we needed the assistive grousers to maintain a constant uniform angle relative to the rover’s body *α* (Figure 3c) independent of the wheel rotation. Our assistive grouser mechanism is able to accomplish this autonomously by virtue of its mechanical design. When needed, the angle *α* can be adjusted using a separate dedicated motor with rotary encoder to measure its angular rotation. As shown in Figure 3c, by measuring the rover body pitch tilting angle *body_tilt* using an accelerometer installed inside the rover body, and *Φ_d_* representing a set target angle for the assistive grousers relative to the gravitational force direction, the angle *α* can be calculated as a function of $\alpha =body\_tilt\;-\;\Phi_{d}$. This function can be then used in an online control loop that allows the assistive grouser to maintain a fixed angle *Φ_d_* relative to the gravitational force direction, regardless of any changes in the body pitch angle that may occur when traversing a steep slope. Setting the angle *Φ_d_* to the known optimal angle for a particular slope angle encountered by the rover is expected to allow the rover to traverse with minimal sinkage. Traversing over a slope with a constant inclination angle means that $body\_tilt\;-\;\Phi_{d}$ will approximate a constant value, reducing the need for the dedicated motor and resulting in near zero electrical consumption by the motor.

### 1.3. Performance Comparison Tests

**Figure 4:**
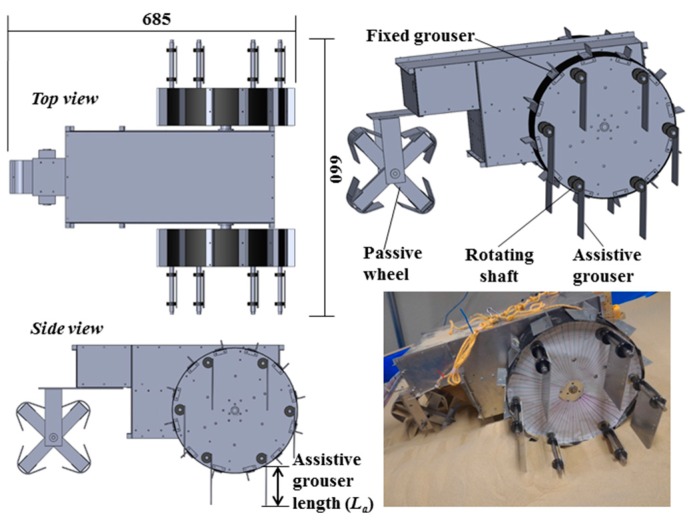
3D CAD drawing and photo of wheeled grouser with attached assistive grouser mechanism.

We carried out comparison performance tests between a rover equipped with a conventional fixed grouser (referred to as a “conventional wheeled rover”) and one with our assistive grouser (referred to as a “modified wheeled rover”) on an indoor sand incline mobility test field developed by us. First, we conducted test runs with the conventional wheeled rover, with grouser lengths set at 20, 40, 60, and 80 mm. The test runs were conducted on sand surface inclination angles of 0 (level incline), 10, 20, and 30 degrees. These tests demonstrated that for conventional fixed grousers, changing the length of the grouser did not effectively reduce the amount of sinkage on any of the sand surface inclination angles. Then, we carried out similar test runs using our proposed assistive grouser. The length of the grouser fixed on the wheel was 20 mm and the assistive grouser mechanism attached to its side. The length of the assistive grouser, *L_a_* (Figure 4), trialed was at 50, 70, and 90 mm, and the absolute angle *Φ_d_* (Figure 3c) was set to be within 60 degrees forward and backward relative to the gravitational force direction. From the test runs, we found that the configuration that produced the least sinkage used the longest assistive grouser length 90 mm, with *Φ_d_* at 20 degrees for 0–10 degrees sand surface inclination, *Φ_d_* at 20–40 degrees for a 20 degrees sand surface inclination, and *Φ_d_* at 40 degrees for a 30 degrees sand surface inclination. In summary, the steeper the sand surface inclination, the less sinkage occurred by setting the assistive grouser angle *Φ_d_* higher.

In addition, by attaching a strain gauge to the assistive grouser and observing the amount of force acting on the grouser during its traversal of the 30 degree sand surface inclination, we observed that for the latter half of the assistive grouser movement towards the back of the wheel, only a small amount of force actually acted on the grouser, confirming the validity of our hypothesis, namely, that the assistive grousers is able to exit the sand surface with little force wasted on lifting sand out from under the wheel.

Mechanisms using similar concept of changing the grouser angle to gain more traction have been introduced, for example by Yang [16] and Hermawan [17], but their performance on sand slope inclines was not tested.

This paper is organized as follows: Section 2 describes the mechanical design and specifications of the rover and explains the experimental setup for this paper; the criteria used to evaluate the traversing performance for the rover are elaborated in Section 3; Section 4 describes the performance comparison experiments carried out between a conventional wheeled rover and a modified wheeled rover. Lastly, in Section 5, the effectiveness of our assistive grouser and its anticipated uses are discussed.

## 2. Materials and Methods

This section describes the modified wheeled rover, the conventional wheeled rover, and the indoor sand incline mobility test field used in this paper.

### 2.1. Wheeled Rover Equipped with Assistive Grousers (Modified Wheeled Rover)

Figure 4 shows the 3D design drawing and a photograph of the modified wheeled rover built for this experiment. The wheel plate, body frame and grousers were made using A5052 aluminum, with the surface of the wheel covered using smooth surface plastic. The front two wheels are driven wheels and the rear wheel is a passive wheel. The passive wheel was fitted with radial ball bearings to ensure smooth rotation. The design concept for the assistive grouser mechanism, as shown in Figure 3, is to act as a supporting device attached to the wheel of a conventional wheeled rover, which operates by reducing the amount of displaced sand during the traversal of sand surface inclines. To avoid interfering with the assistive grouser mechanism when in contact with the sand, the length of the fixed grouser attached to the wheel was chosen to be short (20 mm). It should be noted that most of the conventional wheeled rovers actually in use today are equipped with grousers of a relatively short length. Excluding the length of the assistive grouser and the fixed grouser, the diameter of the wheel was 296 mm, and its width was 90 mm. The fixed grousers attached to the surface of the wheel had 90 mm width, 2 mm thickness, and 20 mm height. There were 12 fixed grousers per wheel, attached at equally spaced intervals. The front wheels were driven by a single DC motor (Maxon Motor Ag, RE40 series, 150 W) attached to a reduction gear (Harmonic Drive Systems Inc., Tokyo, Japan, SHF series harmonic drive SHF-17-100-2UJ) combined with gears and pulleys with a total gear ratio of 156:1, meaning that the front wheels rotates forwards and backwards in sync. The current rover design is unable to turn in different directions, but by installing dedicated driving motors for each wheel, the rover will be able to turn and steer in future designs.

Attached to the outer side of each of the wheels were six protruding rotating shafts placed on radius 130 mm from the center point of the wheel, and on each of the shafts was an assistive grouser with 90 mm width, 2 mm thickness and length *L_a_* of either 50, 70, or 90 mm fixed with clamps and bolts. To improve the strength of the grouser-shaft joint, a key and keyhole combination could be used but for our experiments, clamps and bolts were strong enough. The length of assistive grouser, *L_a_*, was defined as the maximum length of the grouser that protrudes outside the wheel surface during wheel rotation. The number of shafts and the maximum length for the assistive grousers were determined by geometrical constraints. For example, placing six shafts 130 mm from the center of the wheel would only allow clearance space for a maximum grouser length of 90 mm so as not to interfere with the other shafts when the wheel rotates as shown as A in Figure 3c. Reducing the number of shafts might allow for a longer assistive grouser length, but reducing the number of grousers in contact with the sand would reduce the overall traction force generated. The rotating parts of the assistive grouser mechanism were protected from the sand by designing the rover body to be sand-proof, and using radial bearings with dust proof shields.

**Figure 5:**
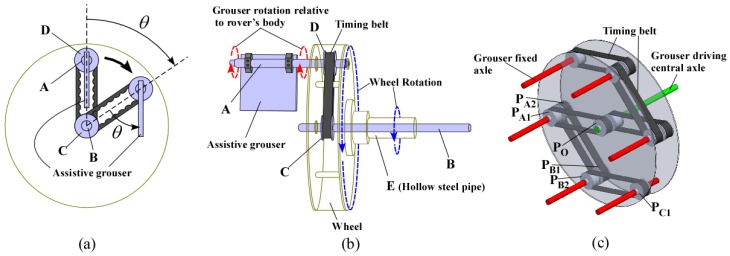
Configuration of the assistive grouser mechanism: (**a**) Side view of wheel rotated *θ* degrees with single assistive grouser; (**b**) Parts of the assistive grouser mechanism; (**c**) Timing belt and pulley arrangement for assistive grouser mechanism.

The assistive grousers were designed to maintain a constant uniform angle relative to the rover’s pitch tilting angle, *α*, independent of the wheel’s rotation. Figure 5 describes the configuration of the mechanism. Figure 5b shows a single wheel with a single assistive grouser attached to it. The shafts A and B were driven in sync by timing pulleys C and D, which was connected using a timing belt. Shaft B was inserted through the hollow pipe E and kept in position using radial bearings. Hollow pipe E was fixed to the wheel. When shaft B and pulley C was not rotated (static relative to the rover body) and the wheel was turned to the blue dotted line direction, pulley D and shaft A will rotate in red dotted line direction. To provide more detail, as shown in Figure 5a, as the wheel was turned clockwise *θ* degrees, pulley D and shaft A would turn counter-clockwise *θ* degrees. Therefore, the angle of the assistive grouser relative to the rover body, *α* could be kept constant relative to the rover body autonomously regardless of the wheel rotation without any additional control. In addition, the angle of *α* could be adjusted to a desired angle relative to the rover body by rotating shaft B. Increasing the number of attached assistive grousers while simply implementing the mechanism as shown in Figure 5b will require an identical number of pairs of timing pulleys C and D for each grouser being installed next to each other, thus increasing the necessary width of the wheel. Therefore, we adopted another design needing only two timing pulleys positioned side-by-side, as shown in Figure 5c. Pulley P_A1_ is connected to central pulley P_O_, which is fixed to the central driving axle; pulley P_B1_ is then connected to pulley P_A2_, which shares the same axle as P_A1_; and then finally pulley P_B2_ connects to pulley P_C1_, connecting the three red axles. The other three red axles are connected in a similar fashion. As a result, all the red axles are driven simultaneously.

In addition to the motor that drives the front wheels, the rover was also equipped with another DC motor (Maxon Motor Ag, RE40 series, 150 W, Sachseln, Switzerland) attached to a reduction gear (Harmonic Drive Systems Inc., Tokyo, Japan, SHF series harmonic drive SHF-17-100-2UJ) and combined with gears and pulleys to have a total gear ratio of 156:1, which was dedicated to adjusting the angle of the assistive grousers relative to the rover’s pitch tilting angle, *α*. We used a high output DC motor, but the electrical consumption of the motor would be relatively small thanks to the mechanism of Figure 5. Therefore, a smaller DC motor could also be used. In addition, by rotating the assistive grousers such that the grousers protrude upwards (angle *α* is controlled so that none of the assistive grouser come into contact with the terrain) when the assistive grouser is not needed, the rover can run in a similar manner to a conventional wheeled rover. As mentioned previously, in a future design where the wheels are driven by dedicated driving motors to enable turning and steering, the assistive grousers will also be driven by separate driving motors for each wheel.

Both the wheel and assistive grouser drive trains used reduction gears with high gear ratios which in combination with the friction inside the drive train, caused the joints to be self-locking and exhibit a high resistance to backward drive force from the load torque from the ground. For the control system for both the wheel and the assistive grouser angles, the angular rotation angle of both motors was measured using the motor’s internal magnetic rotary encoder (Maxon MR Type L, 256 Pulse), and using a feedback control loop, the motor was controlled to follow the incrementally increasing set target angle for the wheel, and a feedback control loop to control the motor to maintain a set target angle *Φ_d_* (Figure 3c) throughout the experiment for the assistive grousers.

**Figure 6:**
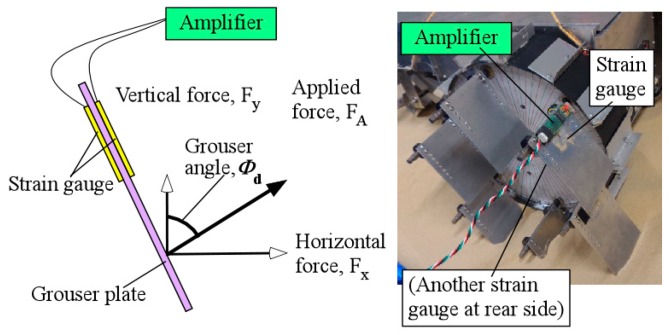
Force measurement using strain gauges attached to grouser.

The sensors used to control and evaluate the performance of the rover were the magnetic rotary encoder (as mentioned above), an accelerometer (Crossbow CXL02LF3, Crossbow Technology Inc., San Jose, CA, USA) to measure the angle of the rover’s body pitch tilting angle relative to the gravitational force direction, and an electrical current sensor (U.R.D. Ltd., Yokohama, Japan, HCS-20-50-AS) to directly measure the value of current flowing inside the cable powering the DC motors. For the experimental evaluation of the assistive grousers in Section 5.1, we used strain gauges (RS Pro, N11MA812023, Yokohama, Japan) to measure the amount of force acting on the assistive grouser only. As shown in Figure 6, the applied force generated from grouser–sand interaction, F_A_, was measured using two strain gauges attached to the front and back sides of the grouser to compensate for measurement error resulting from temperature changes. A strain gauge amplifier was used to amplify the voltage changes in the circuit when force was applied to the grouser. The voltage reading was converted into force by using the recorded voltage-force graph obtained from calibration. Calibration was done by applying force using a force gauge on the grouser and observing the strain gauge circuit output, assuming the amount of strain experienced by the grouser from the total applied force from the changing sand contact area to be similar to the applied force to the center of the grouser plate during calibration. During operation, the assistive grouser angle was continuously maintained at the target angle *Φ_d_* for the assistive grousers relative to the direction of gravitational force (Figure 3c). Using the *Φ_d_* value, the horizontal component F_x_ and the vertical component F_y_ are derived from the applied force F_A_.

To control the DC motors, we assembled our own motor driver circuit board and used a micro robot controller (General Robotix Inc., Tsukuba, Japan, HRP-3P-CN-A), and the 24 V power supply for the motors and the power supply for the sensors, controllers and other devices were placed externally and tethered to the rover by using cables. The weight of the whole rover excluding the externally connected devices was 23 kg.

### 2.2. Wheeled Rover Equipped with Conventional Fixed Grousers (Conventional Wheeled Rover)

The conventional wheeled rover was simply the same rover described above but with the assistive grouser mechanism removed. The length of the fixed grousers was designed to be variable, with 20, 40, 60, and 80 mm as the available options. The number of the fixed grousers attached to each wheel for each of the grouser lengths was 12, identical to the number of fixed grousers attached during the assistive grouser experiments.

### 2.3. Indoor Sand Incline Mobility Test Field

**Figure 7:**
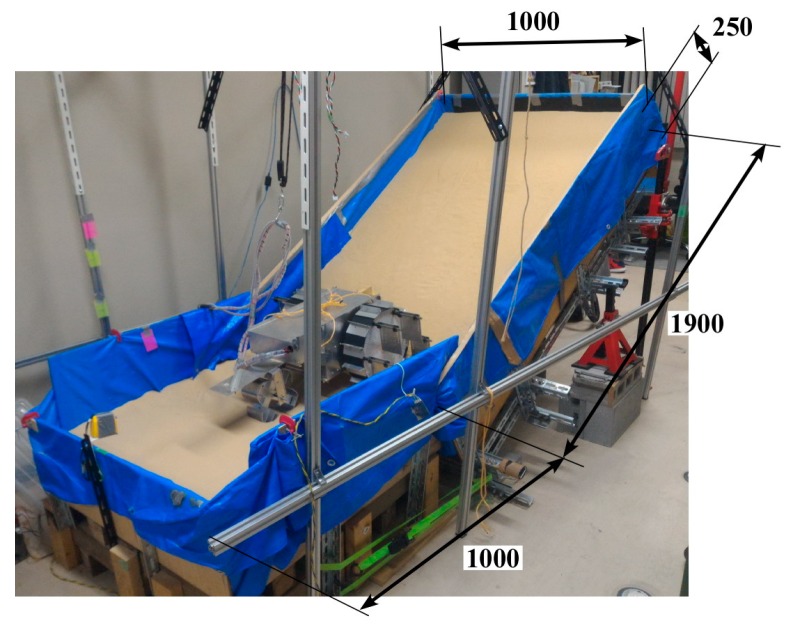
Indoor sand incline mobility test field with sand surface inclination set to 30 degrees.

Figure 7 shows the indoor sand incline mobility test field used to measure the performance of the rovers. The sand box was constructed from wooden boards and steel frames, and it was filled with about 0.725 m^3^ of Toyoura sand in a uniform depth of 250 mm across the surface area, making the total weight of over 900 kg. Obviously, rover mobility is affected by the type of sand used, and we chose Toyoura sand as our test medium because of its homogenous properties that well represent unconsolidated sand terrain. Its geological properties have also been widely investigated [18] and it is widely used in terramechanics research [5,19,20]. The angle of repose (the maximum angle of the slope face created by a flowing granular material when it is piled onto itself before the material slides under its own weight) for dry Toyoura sand has been measured to be an average of approximately 30 (29.71) degrees [10]. As shown in Figure 7, the starting point for the rover was at the end of a 1000 mm long level surface at the foot of the incline, and the rover would start its climb on the 1900 mm long inclined surface. The mobility test would end after the rover touched the board at the top of the incline. Data collection was done after the rover reached a steady running state. The surface condition of the sand changed immediately and rapidly after the rover started the climb from the starting position, as shown in Figure 7, and as a result the data recorded (sinkage angle, sand displacement volume, current consumption, body pitch angle) also became inconsistent. We define the period after the sand surface achieved a consistent state as the steady running state. It differs depending on the slope angle and rover configuration, but in our experiments it was recorded while the rover’s top was between approximately 700 mm and 1900 mm from the foot of the slope. The inclination surface angle of the test field could be set to 0 (level surface), 10, 20, or 30 degrees. The angle of inclination in Figure 7 is 30 degrees.

## 3. Traversing Performance Evaluation

The performance evaluation criterion used in this paper is explained below.

### 3.1. Maximum Wheel Sinkage Angle

**Figure 8:**
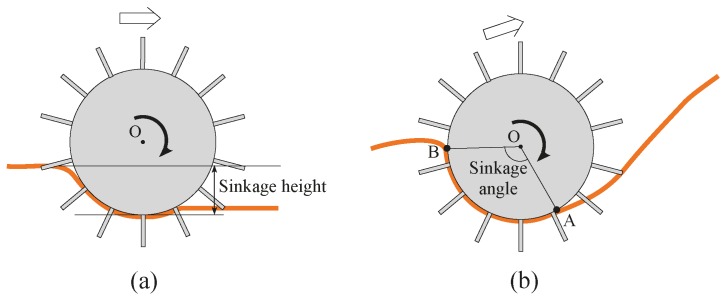
Measuring the amount of sinkage: (**a**) Sinkage height; (**b**) Sinkage angle.

**Figure 9:**
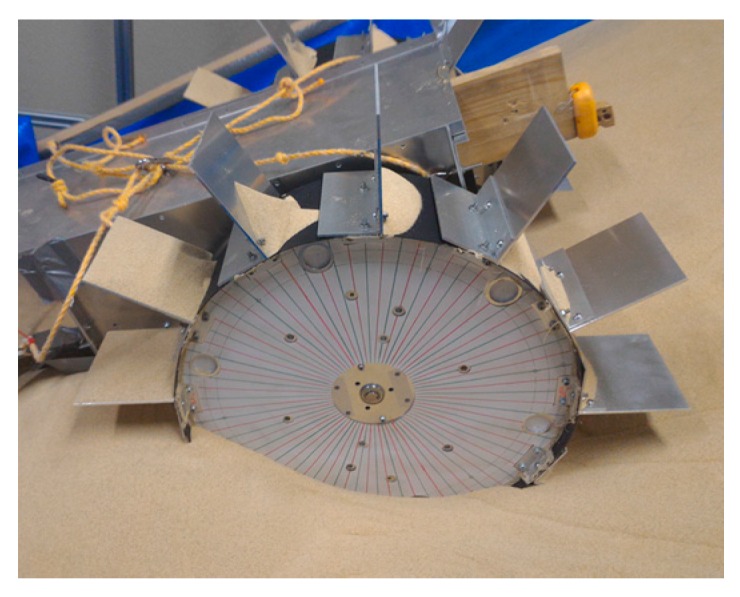
Measurement of the sinkage angle using the angle measurement scale lines. (Sinkage angle for the figure is 130 degrees).

As shown in Figure 8a, when a rover generally traverses a surface made out of soft sand, there is a tendency for the sand to accumulate towards the backside of the wheel as the wheel rotates. The difference in height between the sand surface and bottom of the wheel is defined as sinkage height [11], and the larger the sinkage height, the larger the tendency for the wheel to sink into the sand and enter the “stuck” stage where the wheel is no longer able to recover from its high sinkage condition. This definition is widely applied to level angle sand surfaces, but for steep surface incline angles as shown in Figure 8b, the sand situated in front of the wheel tends to crumble and continuously flow downslope, causing the surface incline to lose its original shape and making it difficult to measure the difference in sinkage height as the rover traverses the incline. Because of this, here, angle AOB from the point of contact between the wheel with the sand at the front side A and the point of contact between the wheel and sand at the back side B, was used as the measurement of wheel sinkage (Figure 8b) and defined as the sinkage angle. The larger the sinkage angle, the larger the sinkage of the wheel into the sand surface and the higher the possibility that the wheel will enter the “stuck” stage. Measuring the location of the points of contact A and B using sensors was difficult, so we recorded video of the wheels as they traversed the slope, and by counting the 5-degree increment angle measurement scale lines stuck to the side of the wheels (Figure 9), the sinkage angle value was visually recorded. During the mobility performance tests, the largest sinkage angle value recorded while the rover was in a steady running stage was defined as the maximum sinkage angle. The maximum sinkage angle is the most important criterion for defining the amount of sinkage and the rate of severity before entering the “stuck” stage; therefore in this paper we treat this criterion with the utmost importance.

### 3.2. Sand Displacement Volume

**Figure 10:**
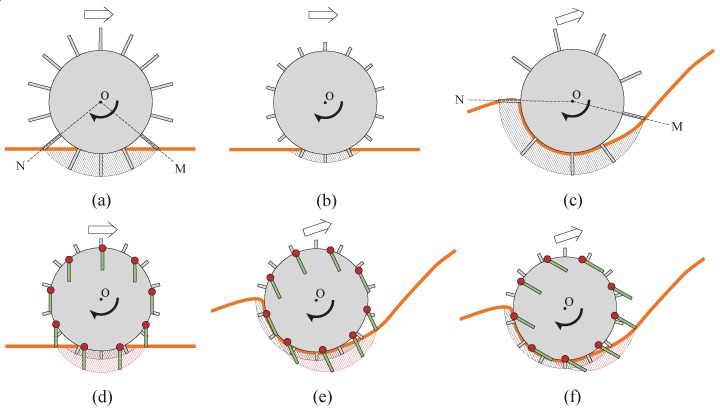
Contact area of the grousers with the sand underneath the surface marked using the shaded area: (**a**–**c**) Conventional wheels; (**d**–**f**) Wheels with assistive grousers.

In this study, we defined the rotation angle $\Theta_{p}$ as the ideal rotation angle needed for a wheel to travel over a set distance without any slipping; rotation angle θ as the real measured rotation angle of a wheel recorded for a wheel to travel over the same set distance. The angle θ was measured using a rotary encoder and $\Theta_{p}$ was calculated by dividing the traveled distance *L* of the rover (as measured by a ruler) by the radius of the wheel, *r*; i.e., $\Theta_{p}=L/r$. The slip ratio [5,21], with a value ranging 1–0, is defined as $1-\left(\Theta_{p}/\theta \right)$. A larger slip ratio means that the wheel experiences a larger amount of slippage, meaning that the traction efficiency is lower and there is a higher tendency for the wheel to enter the “stuck” stage. Lyasko analyzed the effect of slippage on the amount of sinkage experienced by a wheel, and observed a monotonic increase in sinkage for a slip range of 0% to 30%, and a 60% sinkage increase when the slippage was 33% [21]. However for locomotion over soft sand, even if the value of the slip ratios is the same, the differently sized grousers will displace different volumes of sand for the same rotation angle of the wheel. For example, as shown in Figure 10a,b, both wheels have sunk beneath the sand surface, and if both wheels continue to slip for one revolution of the wheel at the same location, the amount of sand displaced by a single grouser could be calculated roughly as the shaded area times the grouser width, and if the grouser widths are the same, the longer grouser will displace a larger volume of sand. A larger sand displacement will cause a larger sinkage and increases the probability that the wheel enters the “stuck” stage. Therefore, we calculated the slip rotation angle using $\left(\theta -\Theta_{p}\right)/360$ in degrees, and we defined the sand displacement volume as the amount of sand volume displaced by all of the grousers attached to the wheel during $\left(\theta -\Theta_{p}\right)/360$ slip rotation angle in degrees. To be more specific, the displaced sand volumes were calculated by multiplying $\left(\theta -\Theta_{p}\right)/360$ with the area of contact for a single grouser (shown as the shaded area in Figure 10) and multiplied by the width of the grouser and then again multiplied by the number of grousers attached to the wheel. Naturally, the area of contact of a grouser (shaded area) would be different for wheels with different amounts of sinkage, even if the grouser length was the same, as shown in Figure 10a,c. By observing the position at which the grouser enters (OM) and exits (ON) the sand, and then calculating the volume of displaced sand between the two positions, the sand displacement volume during the rover’s steady running stage can be calculated.

Similarly, for wheels with assistive grousers, with the sand-grouser contact area shown as the shaded areas in Figure 10d–f, the total sand displacement volume can be calculated by adding the volume of sand displacement caused by the fixed grouser attached to the wheel (black shaded area) and the volume of sand displacement caused by the assistive grouser attached to the side of the wheel (red shaded area). Methods to estimate the volume of sand displaced by the movement of the grousers under the sand surface is available using terramechanic models [22,23,24]; however, the flowing motion of the sand on steep inclines is too complex to accurately model and their models cannot estimate the sand movement caused by our proposed assistive grouser mechanism. The method that we chose to calculate the sand displacement volume is not strictly accurate and may involve some errors, but we believe that the calculation is sufficient to act as a measured criterion in combination with the sinkage angle to determine the probability that a wheel will enter the “stuck” stage. Additionally, the slip ratio is also included as a helpful criterion for evaluating the sand displacement volume.

### 3.3. Current Consumption

Current sensors were used to measure the value of current flowing in the cable that provided electricity supply to the DC motors driving the angular change of the wheel and assistive grousers. The output torque is calculated by multiplying the current value by the torque constant and the gear ratio. For the conventional wheeled rover, only the current consumed by the motor driving the wheel was measured, whereas for the modified wheeled rover, the consumed current measurement considered both the DC motor driving the wheel and that driving the assistive grouser mechanism. As mentioned, however, since the assistive grouser angle relative to the rover body’s pitch tilting angle is mechanically maintained independent of the wheel’s rotation, if the rover is travelling on an incline with a constant inclination angle during the rover’s steady running state, the consumed current will be close to zero. A larger current consumption (output torque) requires a larger actuator to meet the requirements for stable operation, and it requires that the other mechanical parts such as gears, grousers, and bearings are also able to withstand a large stress load, increasing the possibility of mechanical failure in the rover.

### 3.4. Force Applied to a Grouser

By attaching strain gauges to an assistive grouser and observing the amount of strain experienced while traversing a slope, the amount of strain can be translated into an amount of force using the values acquired during calibration of the strain gauge setup. The discussion in Section 5 elucidates the mechanical workings of the assistive grouser beneath the sand surface.

## 4. Results

This section shows the results of the performance comparison experiments carried out. Since wheeled rovers are generally not required to move at high speed and in order to minimize the impact of inertia on the experimental results, the rotation speed for all of the rover configurations was set to be one revolution per minute.

### 4.1. Understanding the Problem by Using a Wheeled Rover Equipped with Conventional Wheels

**Figure 11:**
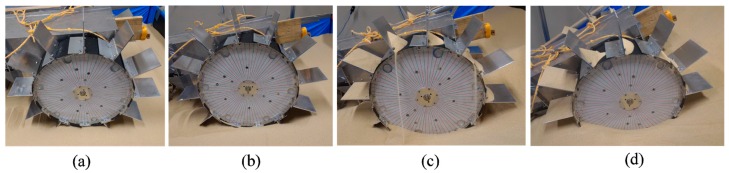
Best performances for conventional wheels with grouser length 80 mm: (**a**) Level incline; (**b**) 10 degree incline; (**c**) 20 degree incline; (**d**) 30 degree incline.

**Figure 12:**
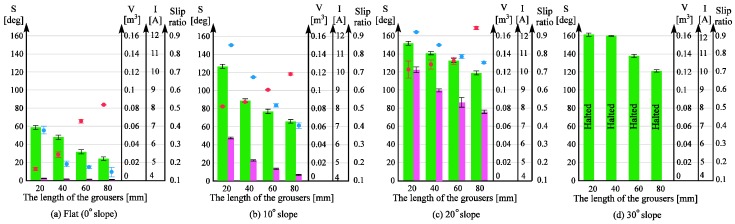
Mobility test results for a conventional wheeled rover on slope inclinations of: (**a**) 0 degrees (level inclination); (**b**) 10 degrees; (**c**) 20 degrees; (**d**) 30 degrees.

The performance of the conventional wheeled rover was observed on a sand surface inclination angle of 0 (level surface), 10, 20, and 30 degrees using the indoor sand incline mobility test field shown in Figure 7. The length of the grouser was 20, 40, 60, and 80 mm. Figure 11a–d shows the pictures taken of the conventional wheeled rover during its best measured performance, and Figure 12a–d shows the measured results for sand surface inclinations 0, 10, 20, and 30 degrees, respectively. On each graph, for the conventional rover with each of the grouser length shown on the horizontal axis, during steady running state, the maximum sinkage angle (the green column), the sand displacement volume per meter of travel distance (the pink column), the slip ratio (the black dot), and the average current consumption (the red dot) are shown as an average value over five runs, and the standard deviation calculated is shown on the graph using the error bar. For all of the experiments, when the consumed current exceeded 20 A, which is much higher than the rated current value for the motor used, the mobility test was stopped to prevent damage to the motors. Although it might be argued that the rover could complete the mobility test if supplied with more than 20 A, we thought that a continued supply of overcurrent was not beneficial in terms of overall energy consumption. The excessive torque caused by overcurrent would also apply an excessive amount of load to the mechanical parts. It should also be noted that for all the experiments carried out in this research, when the consumed current value reached the 20 A limit, the maximum sinkage angle was measured to be over 120 degrees, meaning that the wheel had sunk beneath the sand surface to a large degree and had little possibility of being able to recover, providing another reason for discontinuing the test.

Figure 12a shows the results for mobility tests conducted on a level incline where the force of gravity component parallel to the incline (force C in Figure 1b) does not work against the traction force, and, as described in Introduction, the forward pulling traction force generated by the grouser movement OM-OP (refer to Figure 1a) acts as the dominant force in the system. The graph in Figure 12a shows that a longer grouser length produces a smaller maximum sinkage angle, meaning that the rover traverses the surface with less sinkage. For a grouser length of 80 mm, the maximum sinkage angle recorded was a significantly small value of 24 degrees (Figure 11a). However, as mentioned in the Introduction, a longer grouser length required a larger amount of torque to rotate the wheel, causing a larger current consumption (output torque), as shown in Figure 12a.

Figure 12b shows the results for the moderate incline angle of 10 degrees, where the traction force generated by the grouser movement OM-OP (refer to Figure 1a) is still the dominant force, and this can be confirmed from the results, which show that a longer grouser length still generates a smaller maximum sinkage angle. However, the 10 degree inclination angle generates a small amount of gravity force component parallel to the incline (force C in Figure 1b), which works against the traction force. As a result, the maximum sinkage angle for the 20 mm grouser length was measured at 127 degrees, and for the 80 mm grouser length, the maximum sinkage angle was measured at 66 degrees (Figure 11b), which is relatively larger than when on a 0 degree level incline. The large value of maximum sinkage for the 20 mm grouser length is attributed to the insufficient amount of forward traction force generated by the grouser movement OM-OP (refer to Figure 1a), which caused slippage and allowed the grouser movement OP-ON (refer to Figure 1a) to accumulate a large volume of sand behind the wheel. It should also be noted that, similar to the results for the 0 degree level incline, the average current consumption values were larger when the grousers were longer.

Figure 12c shows the results for mobility tests on the incline angle of 20 degrees, and similar to the above, a longer grouser length generated less sinkage but since the force of gravity component parallel to the incline was larger, for all grouser lengths the maximum sinkage angle and sand displacement volume increased dramatically. The smallest maximum sinkage angle was 118 degrees for the 80 mm grouser length (Figure 11c). The large maximum sinkage angles indicate an unfavorably high probability that the wheel will enter the “stuck” stage. It was also observed that the sinkage angle slowly increased as the rover climbed higher on the incline and that the maximum sinkage angle was recorded when the rover reached the top of the incline. This opens up the possibility that the sinkage angle could further increase as the length of the incline increases. The longer grouser lengths also generated higher average current consumptions (average output torque).

Figure 12d shows the results for mobility tests on the 30 degree incline angle case. For all grouser lengths, the consumed current exceeded 20 A during the initial stage of the mobility test runs and so the runs were halted. The maximum sinkage angle shown in Figure 12d was the value recorded when the mobility test was halted and the rotation of the wheel stopped. The maximum sinkage angle was large enough to indicate a large probability that the wheel would enter the “stuck” stage, but there was also the possibility that the sinkage would continue to increase if the rover was allowed to keep moving. For all grouser lengths, when the rear passive wheels reached the foot of the incline, the rover was unable to travel forward even 10 mm from one revolution of the wheels, which simply spun in the same position. For this reason, the sand displacement volume was not calculated.

The results above are consistent with the explanation shown in Figure 1b. For steep inclines of 20 and 30 degrees, the force of gravity component parallel to the incline (force C in Figure 1b) is large, resulting in insufficient traction force generated during the grouser movement OM-OP (refer to Figure 1a), even when using longer grouser lengths. Increasing the angle OPN increases the amount of sand shifted from under the wheel towards the back, sinking it below the sand surface. On the other hand, using a shorter grouser on an incline angle of more than 10 degrees only generated a small amount of traction force, resulting in a lot of slippage and large amounts of sand displacement, causing sinkage.

From the above results, it can be concluded that for all sand surface inclination angles, a longer grouser length will generate a smaller maximum sinkage angle, but for inclination angles 20 and 30 degrees, the observed sinkage angle even with a longer grouser length was considered to be too large. As such, for a conventional rover using a fixed grouser, it is difficult to determine the optimal grouser length to traverse over all inclination angles from 0 to 30 degrees with minimal sinkage. Furthermore, a longer grouser length requires a larger average current consumption (output torque) regardless of the inclination angle.

### 4.2. Mobility Experiments Using a Wheeled Rover Equipped with Assistive Grousers

#### 4.2.1. Configurations Tested in the Mobility Experiments

**Figure 13:**
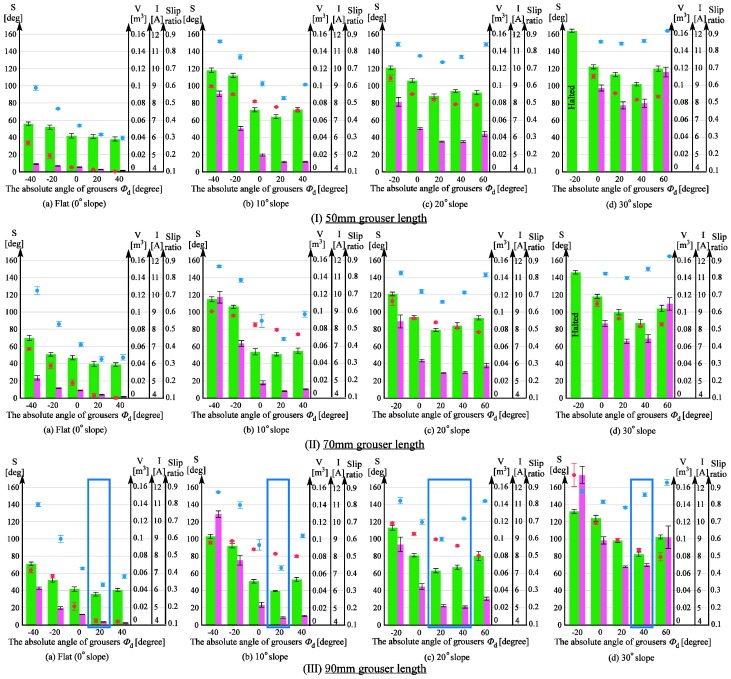
Mobility test results for wheeled rovers equipped with assistive grouser mechanism with grouser length: (**I**) 50 mm; (**II**) 70 mm; (**III**) 90 mm on sand surface inclination angles: (**a**) Level inclination; (**b**) 10 degrees; (**c**) 20 degrees; (**d**) 30 degrees.

In this section, we will verify the ability of a modified wheeled rover to traverse the sand surface incline angles of 0, 10, 20, and 30 degrees with less sinkage compared with the conventional wheeled rover tested above. As shown in Figure 2, the assistive grousers are considered to be more effective when they are longer than the fixed grousers (20 mm), and so the lengths of the assistive grousers used during the mobility experiment were 50, 70, and 90 mm. The results for the experiment are shown in Figure 13. The top row (I), the middle row (II), and the bottom row (III) show the data collected from the experiments with assistive grouser lengths of 50, 70, and 90 mm, respectively. For each length, and similar to Figure 12, the results for 0 (level incline), 10, 20, and 30 degree sand surface incline angles are shown as Figure 13a–d, respectively. The horizontal axis for each of the graphs represents the maintained angle of the assistive grousers relative to the gravitational force direction, *Φ_d_* (Figure 3c) during the mobility tests. For the sand surface inclination angles of 0 degrees (a) and 10 degrees (b), the chosen angle *Φ_d_* = −40, −20, 0, 20, 40 degrees, and for the sand surface inclination angles of 20 degrees (c) and 30 degrees (d), the chosen angle *Φ_d_* = −20, 0, 20, 40, 60 degrees. Similar to the experiments performed in Section 4.1, for each of the configurations, mobility tests were carried out five times and during the steady running state of the rover, the maximum sinkage angle, sand displacement volume per one meter of distance traveled, the slip ratio, and the average consumption current for the five mobility tests’ average were calculated and are represented in the chart as a green bar, pink bar, black dot, and red dot, respectively, with the standard deviation also calculated. If the current consumption exceeded 20 A, the mobility test was halted.

#### 4.2.2. Comparison of Results between Conventional Wheels and Wheels Equipped with Assistive Grouser in the Optimal Configuration

For each sand surface inclination angle, the length of the assistive grouser and the assistive grouser angle *Φ_d_* that produced the smallest maximum sinkage angle was designated as the optimal configuration for that sand surface inclination angle. The optimal configurations for each sand surface inclination angle are marked in Figure 13 by a blue rectangle (for Figure 13IIIc) both *Φ_d_* = 20 and 40 produced similar maximum sinkage angles and sand displacement volumes, and so both of them were designated as the optimal configuration). By comparing the performance of the rover when using the optimal configurations with the results discussed in Section 4.1, we can observe a noticeable improvement.

**Figure 14:**
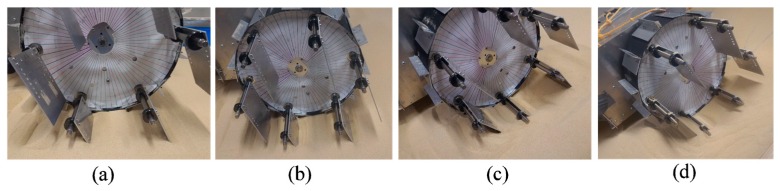
Modified wheeled rover with assistive grouser attached using the optimum configurations: (**a**) *Φ_d_* = 20 degrees at level incline; (**b**) *Φ_d_* = 20 degrees at 10 degree incline; (**c**) *Φ_d_* = 40 degrees at 20 degree incline; (**d**) *Φ_d_* = 40 degrees at 30 degree incline.

For 30 degree sand surface inclination angles, the conventional wheeled rover was unable to climb the inclination. However, the modified wheeled rover using the configuration marked by the blue rectangle in Figure 13IIId, where the assistive grouser length was 90 mm and the angle *Φ_d_* was 40 degrees forward, the smallest maximum sinkage angle observed during steady running was 82 degrees (Figure 14d) and the rover successfully climbed the inclination. The average current consumption during the experiment was measured as 8.36 A, significantly less than that of the conventional rover, which exceeded 20 A.

Similarly for the inclination angle 20 degrees, the conventional wheeled rover exhibited maximum sinkage angles over 118 degrees regardless of the rover’s configuration. The modified wheeled rover in the optimal configuration as shown in Figure 13IIIc (assistive grouser length 90 mm and *Φ_d_* 20 and 40 degrees), the maximum sinkage angles observed were 63 degrees and 67 degrees, showing obvious improvement. In addition, for the conventional wheeled rover, the smallest sand displacement volume was 0.076 m^3^ for a fixed grouser length of 80 mm, but for the modified wheeled rover, in the optimal configuration shown in Figure 13IIIc, the sand displacement volume was only 0.022 or 0.21 m^3^, also a notable improvement. Comparing the average current consumption, the conventional wheeled rover showed a minimum average current consumption of 10.1 A for the fixed grouser length of 20 mm, but the modified wheeled rover in the optimal configuration shown in Figure 13IIIc consumed as little as 8.6 or 9.0 A. In Figure 13IIIc, *Φ_d_* = 20, 40 can be seen to have resulted in the smallest maximum sinkage angles and sand displacement volumes, and so *Φ_d_* between 20 and 40 degrees is considered to be the optimal configuration.

For sand surface inclination angles of 20 and 30 degrees, the sinkage angle of the conventional wheeled rover gradually increased, and therefore, the maximum sinkage angle was recorded when the rover reached the top of the incline or when the rover halted because of excessive current consumption. This leaves open the possibility that the sinkage angle would have continued to increase with longer incline lengths or larger output torques. In contrast, the modified wheeled rover exhibited maximum sinkage angles in the middle of the climb, and this angle remained nearly constant until the end of the experiment. Therefore, it can be assumed that the wheeled rover with the assistive grouser is able to maintain low levels of sinkage even on longer inclines.

For the moderate sand surface inclination of 10 degrees, the optimal configuration from Figure 13IIIb (assistive grouser length: 90 mm, *Φ_d_*: 20 degrees forward) showed a maximum sinkage angle of 40 degrees, much smaller than the maximum sinkage angle of 66 degrees for the conventional wheeled rover with grouser length 80 mm. In addition, the average current consumption for the optimal configuration shown in Figure 13IIIb was 8.14 A, smaller than any of the values measured for the conventional wheeled rover, as shown in Figure 12b.

For the level surface incline, the optimal configuration shown in Figure 13IIIa (assistive grouser length: 90 mm, *Φ_d_*: forward 20 degrees) showed a maximum sinkage angle of 36 degrees and sand displacement volume of 0.0037 m^3^, which is inferior to the best result obtained for the conventional wheeled rover as seen in Figure 12a (sinkage angle: 24 degrees; sand displacement volume: 0.0009 m^3^). However, a maximum sinkage angle of 36 degrees is still considerably small and the difference in the probability of entering the “stuck” stage is not considered. On the other hand, the conventional wheeled grouser with 80 mm fixed grouser length consumed an average current of 8.19 A, almost double compared with that of the optimal configuration shown in Figure 13IIIa (4.24 A).

To conclude, the modified wheeled rover demonstrated the best performance on all sand surface inclination angles with an assistive grouser of length 90 mm, and the angle of the assistive grouser relative to the gravitational force direction *Φ_d_* that produced the best performance was 20 degrees for a 0–10 degrees inclination, 20–40 degrees for a 20 degrees inclination angle, and 40 degrees for a 30 degrees inclination angle. In short, by using a rover equipped with assistive grousers of length 90 mm and adjusting the angle of *Φ_d_* according to the angle of the inclination the rover is traversing, it will be able to travel on all soft sand surface inclinations. Furthermore, the average current consumed by the modified wheeled rover was smaller, meaning that less torque output is required to rotate the wheels, and so the size of the actuators can be reduced. This would also reduce the damage to the mechanical components such as grousers and gears when traversing over steep inclines.

#### 4.2.3. Performance Comparison of the Assistive Grouser with Different Grouser Lengths

This section discusses the effect of grouser length on the performance of the rover for the same sand surface inclination angle and the same *Φ_d_* in Figure 13. For surface inclination angles 10, 20, and 30 degrees, as the length of the assistive grouser increased I→II→III (Figure 13), the maximum sinkage angle and sand displacement volume decreased overall. Similarly, for a level (0 degree) incline, a longer assistive grouser did lead to smaller sinkage angles, but the difference between the values was not so noticeable, and similarly for sand displacement volume and average current consumption. This can be explained by the gravitational force component (force C in Figure 1b) not working against the traction force on level inclinations, allowing even the shortest grouser length (50 mm) to generate enough traction force to travel forward.

For conventional wheeled grousers, a longer grouser length showed an obvious increase in average current consumption, but for modified wheeled rovers, an increase in the assistive grouser length did not show a similar increase. For example, on the 30 degree surface incline angle in Figure 13, assistive grouser lengths 50, 70, and 90 mm with the optimal *Φ_d_* angle of 40 degrees had associated current consumptions of 8.18 A, 8.16 A, and 8.36 A, respectively, showing that an increase in grouser length did not dramatically change the average current consumption. As mentioned in the Introduction, when the length of the attached fixed grouser is increased on a conventional wheel, after the grouser contacts the sand (OM in Figure 1b), the moment arm *l* increases in length, necessitating a larger torque to rotate the wheel. On the other hand, the assistive grousers enter the sand with a piercing motion, reducing the length of the moment arm (*l* in Figure 2b), requiring less torque to rotate the wheel and a lower current consumption. Therefore, based on the results obtained using the above-mentioned configurations, we discovered that longer assistive grousers could lead to a lower probability of becoming stuck while consuming only a relatively small amount of current.

#### 4.2.4. Performance Comparison of Rovers with Different Assistive Grouser Angles

This section discusses the effect of the assistive grouser angle relative to the gravitational force direction, *Φ_d_*, on the performance of the rover. As seen in Figure 13, adjusting *Φ_d_* to be “more forward” (*Φ_d_* > 0) than the gravitational force direction (*Φ_d_* = 0) resulted in lower levels of sinkage, and as the sand surface inclination angle became steeper, adjusting the angle of the assistive grousers further forward (i.e., a larger value of *Φ_d_*) was observed to improve the performance of the rover (be advised that in Figure 13, the values on the horizontal axis for (a), (b) and (c), (d) are different). However, tilting the angle of the assistive angle beyond the optimal angle as seen in Figure 10f reduced the volume of sand in contact with the assistive grousers, and thus reduced the traction force generated, increasing slip and the sand displacement volume. This is especially so on inclined sand surfaces. On the other hand, adjusting the grouser angle backwards relative to the gravitational force direction (*Φ_d_* < 0) will cause the assistive grouser motion to lift the sand from below the surface and allow it to accumulate at the back of the wheel, and the amount of traction force will be also reduced. Clear evidence for this can be seen in Figure 13, where the leftmost side bar shows the largest maximum sinkage angles and the largest displaced sand volumes. For assistive grouser lengths of 50 and 70 mm on a 30 degree sand surface inclination (Figure 13Id,IId) and *Φ_d_* = −20, the rover wheel experienced slipping and did not manage to travel forward as well as the experiment was halted as the current consumption exceeded 20 A.

From the results discussed above, adjusting the assistive grouser angle forward resulted in lower levels of sinkage when traversing sandy inclines, consistent with the concept described in Figure 2. As for average current consumption, the *Φ_d_* that resulted in the smallest maximum sinkage angle also showed the smallest average current consumption.

#### 4.2.5. Relationship between Slip Ratio and Sand Displacement Volume

Referring to Figure 13, both the modified and conventional wheeled rovers exhibit a similar trend of larger sand displacement volume when the slip ratio is higher (i.e., a higher amount of slippage). However, as discussed in Section 3.2, a lower slippage when climbing a slope does not necessarily imply a smaller sand displacement volume. As shown in Figure 13, when using longer assistive grouser lengths and a large angle *Φ_d_* (i.e., the assistive grousers are angled forward) on steep slopes, even though the slip ratio is relatively high, the sand displacement volume does not significantly increase (e.g., *Φ_d_* = 40 for IIIb, *Φ_d_* = 40 and 60 for IIIc, *Φ_d_* = 40 and 60 for IIId in Figure 13). It can be inferred that this is because the assistive grousers minimally displace sand from under the wheel to the back of the wheel when the grouser angle is angled largely forward, as shown in Figure 10f.

## 5. Discussion

### 5.1. Assistive Grouser Effectiveness

For a conventional wheeled rover with grousers attached to the wheel, when enough traction force is generated to compensate for the force of gravity component parallel to the incline surface, the amount of slip is reduced and the rover is able to climb a sand surface incline with minimal sinkage. When there is not enough traction force generated, however, the amount of slip will increase and as the wheel continues to rotate, it will lift sand from below the surface increasing sinkage and accumulating sand behind the wheel. As the sand surface inclination angle increases, the volume of sand in the shaded area between OP and ON increases (see Figure 1b). Furthermore, as the length of the grouser increases, the ratio of the volume of sand in the shaded area between OP and ON to the area between OM and OP increases. Therefore, attempting to increase traction forces generated by the grouser movement OM-OP by increasing the length of the grouser will tend rather to increase the amount of sand lifted by the grouser movement OP-ON. Consequently, as shown in Figure 12c,d, on 20 and 30 degree angle inclinations, an increase in the length of fixed grousers did not reduce the maximum sinkage angle. In addition, as the length of the fixed grousers increased, a larger torque was required to rotate the wheel when it contacted the sand surface Figure 1b OM, leading to a higher output requirement for the actuator, and increased load stress on the parts of the rover, reducing its durability.

**Figure 15:**
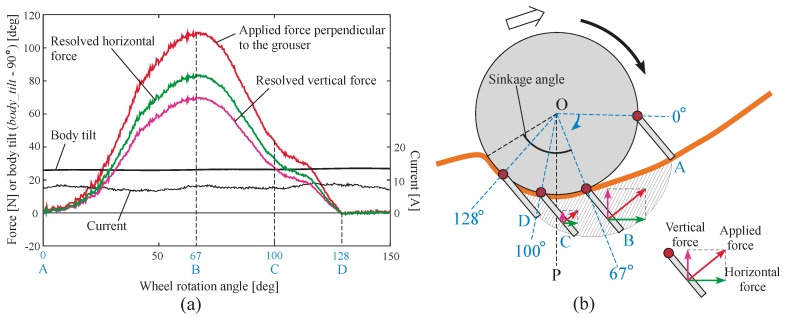
Results from the experiment to validate the effectiveness of assistive grousers on a sand incline of 30 degrees: (**a**) Sequential ordered graph of measured data; (**b**) Different states of a single assistive grouser as it moves under sand surface.

To solve these problems on sand surface inclines, we proposed the use of assistive grousers that could generate a large amount of traction force during grouser movement OM-OP, and exit the sand surface during grouser movement OP-ON without displacing large amounts of sand (see Figure 2b). The assistive grousers are attached to the side of a conventional wheel of a rover as an option. To verify the efficacy of our assistive grouser mechanism, we examined data obtained during the steady running condition in one of the mobility tests with the optimal configuration for the modified wheeled rover on an inclination angle of 30 degrees as shown in Figure 13IIId (assistive grouser length: 90 mm; *Φ_d_*: 40 degrees), and a sequential ordered graph of the measured data is shown in Figure 15a. Figure 15b displays the state of a single assistive grouser as it is moving under the sand surface. The angular position of the grouser with the sand (marked as A in Figure 15b) was set to 0 degrees, and the sequential order of the wheel rotation angle from 0 to 150 degrees was set as the horizontal axis of the graph in Figure 15a. Then, corresponding to the wheel rotation angle, the force acting perpendicular to the grouser surface (the red line), the vertical (pink line) and horizontal (green line) components of the force were measured using strain gauges attached to the assistive grouser and plotted on the graph (see Section 2.1 for detailed information on the strain gauges). In addition, the graph shows the pitch tilting angles of the rover body with the horizontal line set as the zero angle line and upward tilting line representing a positive output in degree (*body_tilt*—90 degrees in Figure 3c). The consumed current is also shown in Figure 15a. The direction of the three force components acting on an assistive grouser is shown in the lower right of Figure 15b. In Figure 15a, the four points marked in blue, A (0 degrees), B (67 degrees), C (100 degrees), and D (128 degrees), correspond, respectively, to the A, B, C, and D marked grouser positions during wheel rotation in Figure 15b. Looking at the graph, until approximately 20 degrees of rotation after the grouser came in contact with the sand at position A, only a small amount of force acted on the grouser because of its piercing movement into the sand surface. After that, the forces acting on the grouser gradually increased until reaching the peak at position B. This is the duration when the grouser was acting as a strong anchor inside the sand surface. The force acting on the grouser gradually decreased until position C, and especially when the grouser moved past the line OP from position C to D, where the acting force was largely reduced. After reaching position D, the force measured on the grouser was almost zero even though the grouser was still in contact with the sand. This was because the grouser movement after the line OP was working solely to pull the grouser out of the sand surface. For conventional fixed grousers, it can be speculated that the grouser movement between point B and point D would generate a large force acting on the grouser. This is the primary cause of the lifting of sand from underneath the wheel to the surface. For our assistive grouser, the measured vertical component force always pointed upwards, meaning that the grouser was constantly pushing down on the sand and gaining traction while also not lifting the sand from underneath the surface past the line OP.

### 5.2. The Effects of Other Wheel Configurations on Modified Wheeled Rover Performance

Based on the experiments performed in Section 4.1 and Section 4.2, it is considered that the longer the assistive grouser is, the more effective it will be as an anchor, preventing slippage of the wheel on steep sand inclinations. In addition, because of the assistive grousers’ piercing and extracting motions, increasing the length of the assistive grouser did not dramatically increase the consumed current (the output torque).

Based on this result, it could be assumed that longer assistive grousers are more effective. However, it should be noted that the experiments in Section 4.1 and Section 4.2 were carried out with a fixed configuration of number of assistive grousers, rover weight, wheel diameter, and wheel width, and the results obtained were limited to specific conditions. Thus, we are unable to confirm the assumption that increasing grouser length is generally more effective. For example, the number of assistive grousers fixed on each wheel was six, but Liu et al. have pointed out that the number of grousers and the spacing between them have a significant effect on drawbar pull, driving torque, and steering resistance moment [25]. Bauer et al. also reported a 30% increase in drawbar pull for their wheel configuration when the number of grousers fixed to the wheel was doubled from 9 to 18 [26]. According to Skonieczny et al., an inadequate number of grousers on a wheel induces forward flow of sand ahead of the wheel as it rotates, increasing a forward rolling resistance working against the traction forces [27]. Therefore, even for our modified wheeled rover, increasing the number of shorter assistive grousers could generate better performance.

In addition, unlike in conventional fixed grousers, the space between the assistive grousers continuously changes as the wheel turns. For example, as shown in Figure 15b, if the grousers are fixed on points B, C, and D at equal intervals on the wheel, we can see that the space between grousers C and D is smaller than that between grousers B and C. Because of this characteristic, the assistive grousers beneath the sand surface will compress the sand volume between the grousers during the latter half of grouser contact with sand, and expand the sand volume between the grousers during the first half of grouser contact with sand beneath the sand surface. We predict that the effect of this sand–grouser interaction will be larger when the number or length of the assistive grousers is increased. It is important to evaluate the effect of this particular sand-assistive grouser interaction on overall rover running performance in future work. Similarly, the effect of rover weight, wheel diameter, and wheel width also affect the performance of a rover wheel [5], so it is important to analyze the effects of these parameters on the running performance of our modified wheeled rover in the future.

### 5.3. Expected Issues and Future Work for Use in a Natural Environment

In practice, the use of our proposed mechanism in a natural environment is expected to produce various problems and their corresponding measures. These are discussed in this section.

#### 5.3.1. Climbing a Slope with Non-Uniform Inclination Angles

**Figure 16:**
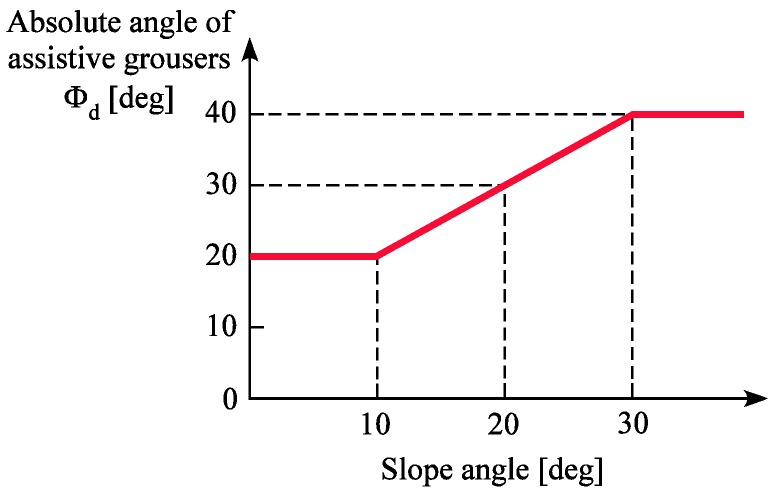
Proposed method of setting the target angle *Φ_d_* (Figure 3c) in a real environment.

The slope face of an inclination in a natural environment will be different from that of the test bed, as the slope’s surface angle will not be uniform, but rather vary randomly depending on the terrain conditions. As a solution, we propose continuously changing the angle of the assistive grousers according to the current slope angle. A conventional wheeled rover is not able to change the angle of its grouser during motion. In contrast, if the angle of terrain inclination can be measured using external sensors or estimated by measuring the tilting angle of the rover body itself in an actual environment, our modified rover will control *α* (Figure 3c) for *Φ_d_* to be optimal to ensure the least amount of sinkage over various degrees of inclination, which is an implementation of both the elements of sensing and grouser control. Based on the experiments in Section 4.2, the ideal length for all inclination angles was found to be 90 mm. The ideal angle of *Φ_d_* for a 0–10 degrees inclination was found to be 20 degrees, for a 20 degrees inclination, it was between 20 and 40 degrees, and for a 30 degrees inclination, it was 40 degrees. Therefore, in a real environment, as shown in Figure 16, by setting the vertical axis value of angle *Φ_d_* as the target angle for controlling *α* depending on the horizontal axis value of the measured slope angle, we believe that traversing any slope angle with minimal sinkage is possible using our proposed assistive grouser mechanism. Obviously, terrain in natural environments also incline in the frontal plane, so in the future we will conduct experiments to test the performance of our modified wheeled rover in such situations.

#### 5.3.2. Deploying and Stowing the Assistive Grousers

Wheeled rovers with installed assistive grousers are built to function as wheeled rovers with conventional wheels simply by rotating the assistive grousers away from the surface of the terrain. However, deciding which situations the assistive grousers should or should not be deployed and the specific method to deploy and stow the grousers in a natural environment is an important challenge for future work.

Based on the results in Section 4.2, superior performance could be obtained using 90 mm long assistive grousers to traverse all angles of sandy inclination. Therefore, when travelling only on sandy surfaces, it can be assumed that there is no need to stow the assistive grousers upwards, and that there is no need to attach fixed grousers to the wheel when assistive grousers are used. It has already been confirmed that with either no fixed grousers or with fixed grousers of 10 mm length, the rovers are able to traverse over all sand surface inclinations demonstrating performance results similar to Figure 13. To ensure that the fixed grousers do not interfere with the working of the assistive grousers, the length of the fixed grouser should be minimized. However, if it is possible that a scenario may arise in which not using the assistive grouser mechanism is preferable, such as a descending steep slope or travelling over solid surfaces, attaching fixed grousers of short length is the better alternative.

We expect that switching between deploying and stowing the assistive grousers will only be required when the terrain conditions change significantly, meaning that there should be no need for it to be done frequently. However, deploying and stowing the assistive grousers while still on a sand surface will cause plowing of the sand as a result of the grouser’s angular movement, generating resistance on the grousers. Therefore, a method of switching that reduces the load on the grousers—for example, by deploying the assistive grousers slowly while turning the wheel—should be investigated in the future.

#### 5.3.3. Running on Terrain with Different Soil Types

The experiments in this paper used Toyoura sand, a typical fine sand, but if the assistive grouser were to be used on a stiffer surface made out of another type of sand, there is a possibility that the 90 mm length would not fully pierce the sand surface, causing the wheel to be lifted off the terrain as the wheel rotates. Assistive grousers with shorter lengths were proved to be able to act as anchors and allow the traversing of sand slopes with low levels of sinkage as well, and so we believe that the type of sand traversed should be considered when determining grouser length. Furthermore, verifying the effectiveness of our proposed concept on surfaces with different properties other than Toyoura sand will be the subject of our future work.

#### 5.3.4. Turning and Steering

In future work, the current test rover design will be modified to allow turning and steering. Among the most common methods to steer wheeled rovers are using differential steering (steering by the difference in angular velocities of the wheels) and active steering using dedicated steering motors on each wheel [28]. Generally, both steering methods induce a yaw rotation of the grousers inside the sand with respect to a fixed ground frame, generating steering moment resistance on the surfaces of the grousers.

Our modified wheeled rover uses assistive grousers with a large contact surface area and generate a non-trivial amount of resistance if the steering radius is small, obstructing the steering of the rover. When on a hard surface or on a soft surface with minimal sinkage, the assistive grousers can be stowed away and steering performed in a similar manner to conventional wheeled rovers. However, if the surface is soft enough to cause more sinkage, then, even if the assistive grousers are stowed upward, then the sinkage will still cause the sand to come in contact with the stowed grousers and generate resistance during steering. Therefore, compared to conventional wheeled rovers, the modified wheeled rover is not as suitable for carrying out steering maneuvers with small steering radius.

However, to cope with the possibility of the rover needing to perform a steering maneuver with small steering radius (during an emergency, for example), in future work a differential steering method will be applied on the modified wheeled rover so that it can make use of its high-powered driving motors to overcome the steering resistance forces while steering.

Steering while on a slope of soft sand is difficult because of the larger amount of slippage [28]. In our modified wheeled rover, steering can be achieved by artificially inducing slippage for a single wheel while maintaining traction on the other, by stowing away the assistive grousers on a single wheel and leaving the assistive grousers on the other wheels in the ground, if separate motors are used to manipulate the assistive grouser angles on each wheel. This might allow for easier steering on a steep slope, and this can only be achieved using a stowable grouser mechanism such as ours.

To implement a fully independent drive for the left and right wheels requires having not only a dedicated wheel driving motor but also a dedicated grouser angle driving motor for each wheel. However, since only a small motor is required for driving changes in grouser angle, this may reduce the downside of increasing the total number of actuators.

#### 5.3.5. Durability of Longer Grousers

**Figure 17:**
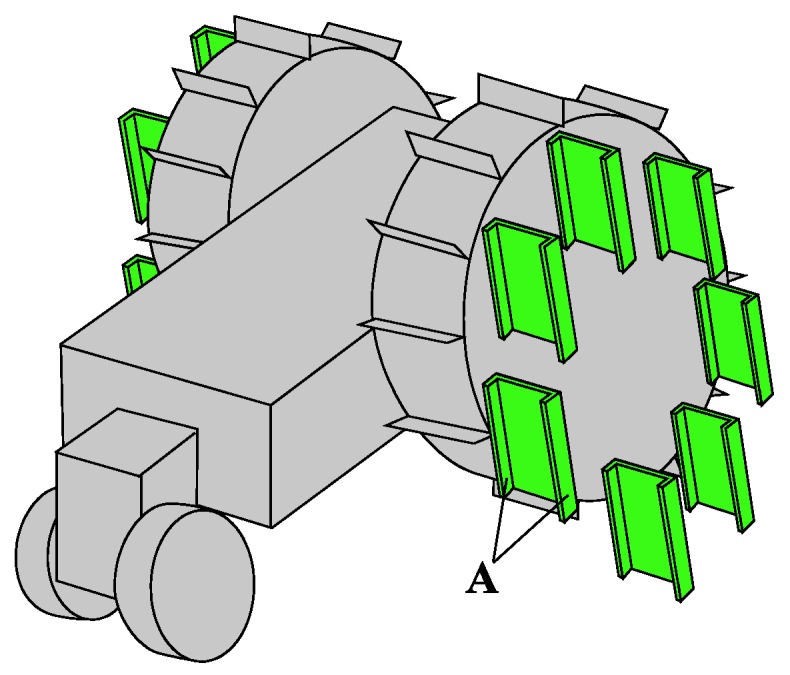
Channel shaped assistive grousers.

Based on our experimental results, a longer assistive grouser length is believed to be more effective, but the prolonged use of a long grouser might have issues with material strength and durability. To address that problem, we are considering grousers made using a channel shaped material such as that shown in Figure 17. The addition of side walls “A” will trap and stop the flow of the sand to the sides of the grouser, generating a larger traction force and increasing the effectiveness of the grouser as an anchor, and also during the pulling action of the grouser during the movement OP-ON (Figure 2b). The side walls “A” do not inhibit the pulling action of the grouser from beneath the sand surface. In contrast, if the fixed grousers used on conventional wheels are made using channel shaped material, the volume of sand lifted during the grouser movement OP-ON would increase.

## 6. Conclusions

In order to achieve the goal of climbing a steep slope of soft sand without displacing a large amount of sand, we proposed a concept maintaining a constant grouser angle independent of wheel rotation. To verify the proposed concept, we assembled a modified wheeled rover with the proposed grouser mechanism attached to the side of a conventional rover wheel, and carried out tests in comparison with a conventional wheeled rover with fixed grousers.

Through the experiments, we found that the modified rover was able to traverse all the tested sand surface inclination angles (0, 10, 20, and 30 degrees) with lower levels of sinkage as the length of the assistive grousers was increased. By way of comparison, increasing the length of the fixed grousers in a conventional rover did not assist it in climbing the steeper sand surface inclinations and it could not even traverse the 30-degree inclination.

However, it should be noted that these conclusions are based on the results of experiments where parameters other than grouser angle and length (e.g., number of assistive grousers, grouser width, weight of rover, wheel width, and wheel diameter) were fixed for all climbing runs. Furthermore, the experiments were carried out in limited environments using a single type of sand condition, with the initial sand slope angle set to be a uniform angle without a roll angle inclination. Therefore, further detailed validations of our proposed concept will need to be performed in future work by incorporating more variable rover design parameters. For usage in a natural environment, there are design challenges (as discussed in Section 5.3) that will also need to be addressed in future work. Moreover, mobility tests will be conducted outdoors on real sandy terrain, such as in the desert. An investigation of the effectiveness of increasing the number of driven wheels equipped with our assistive grouser mechanism to four or six is also planned.
